# Enhancing EFL students’ critical thinking skills using a technology-mediated self-study approach: EFL graduates and labor market in perspective

**DOI:** 10.1371/journal.pone.0293273

**Published:** 2023-10-19

**Authors:** Sami Algouzi, Ali Abbas Falah Alzubi, Mohd Nazim

**Affiliations:** Department of English, College of languages and Translation, Najran University, Najran, Saudi Arabia; Management and Science University, MALAYSIA

## Abstract

This research project bridges the gap between Saudi Vision 2030 and labor market needs by strengthening English as a Foreign Language (EFL) students’ critical thinking skills. The increasing unemployment rates may not be due to insufficient vacancies in the labor market, but graduates’ lack of the general abilities deemed vital to meet the labor market needs. With employability in mind, this study reiterates that graduates should ideally be advanced specialists, critical researchers, creative initiators, and active communicators to be more competitive and contribute to the prosperity of their nation. Therefore, this research employs a quasi-experimental design (time series design) to investigate how effectively students’ critical thinking skills are enriched using a video-mediated self-study program through Telegram. Studies in this respect, regarding the Saudi EFL context, are limited. Therefore, this research employed a video-mediated self-study program through Telegram on learning critical thinking skills for EFL students majoring in English or Translation. The data collection included a pre-and post-test on critical thinking skills and a semi-structured interview. The findings showed that students improved their critical thinking skills due to the training program compared to their performance before the treatment at a low level. Besides, the participants evaluated learning critical thinking skills from thinking ways, feelings, benefits, motivation, challenges and problems, and suggestions. In light of the findings, recommendations were presented.

## Introduction

Critical thinking is a highly demanded skill nowadays. It is one of the most important talents to develop for future success. Being critical of everything is what critical thinking entails. It comes down to objectivity and keeping an open mind. To think critically is to assess topics based on authentic information rather than personal beliefs and biases. Critical thinking assists people in developing deep knowledge that allows them to make better decisions and handle issues more efficiently. When addressing issues and making decisions that affect ourselves, our families, our country, and our planet, we all require critical thinking abilities and habits of mind. Learning necessitates critical thinking skills since it necessitates the interpretation and integration of new information, as well as its practical and suitable application when confronted with fresh scenarios, issue conditions, and inventive chances [1]. In other words, critical thinking is coming to your well thought conclusions rather than accepting facts at face value [2].

Education should adapt to the needs of the labor market. In this regard, Saudi Vision 2030, being a unique transformative economic and social reform blueprint, identifies the ways to enrich the services offered by the sectors like health, infrastructure, recreation, tourism, and education. Several fundamental concepts are presented, such as the attributes of university graduates who possess critical thinking skills to meet the labor market needs and make education in the Kingdom of Saudi Arabia a leading example. Numerous reports in various publications imply that Saudi university graduates struggle to find employment opportunities [3–5]. The reports suggest that the increasing unemployment rates may not be due to insufficient vacancies in the labor market, but graduates’ lack of the general abilities deemed vital to meet the labor market needs, especially in light of the national Saudization program.

With employability in mind, graduates should ideally be advanced specialists, critical researchers, creative initiators, and active communicators to be more competitive and contribute to the prosperity of their nation. Therefore, recent years have witnessed numerous innovative instructional models for developing English as a Foreign Language (EFL) learners’ learning skills, including critical thinking skills. Researchers have conducted various experiments to explore, examine, and assess the effectiveness of many methods, approaches, and strategies and suggested many pedagogical models to strengthen EFL students’ learning skills. There are reasons for implementing critical thinking in language education. Atkinson [6] argues that critical thinking is a social practice that everybody can learn and practice in any environment unconsciously. Also, Afshar and Movassagh [7] claim that by learning critical thinking skills, students do not take things for granted and respect for intelligent criticism and direct statement. In addition, a plenty of studies cited a bilateral connection between critical thinking and foreign language learning [8–15].

In addition, the utilization of digital practices has the potential to be very interesting and helpful because they integrate very efficient activities and are of interest to students. Nevertheless, the synthesis of literature highlights a lack of research investigating the impact of a video-mediated self-study program through Telegram to boost EFL students’ capabilities to think critically. The significance of the current study lies in blending utilizing a video-mediated self-study program through Telegram to strengthen EFL students’ critical thinking skills. Therefore, this study, while supporting earlier studies that investigated critical thinking skills in various contexts, intends to investigate how well a video-mediated self-study program through Telegram works at improving EFL students’ critical thinking skills. The study is aimed at

1. investigating how a video-mediated self-study program through Telegram would improve EFL students’ critical thinking skills.
2. examining students’ learning experience and attitude toward critical thinking skills using a video-mediated self-study program through Telegram.

## Theoretical framework

### Critical thinking

Facione [16] defined critical thinking as “the process of purposeful, self-regulatory judgment. This process gives reasoned consideration to evidence, context, conceptualizations, methods, and criteria.” This definition entails the mental talents and habits related with critical thinking strength. Critical thinking is the process of making deliberate, reflective decisions about what to believe or do. Critical thinking is an all-encompassing human phenomena; people examine information, understand events and circumstances, and evaluate assertions and the explanations presented to support them several times every day. Also, they draw conclusions and make thoughtful decisions about what to believe and what to do based on such analyses, interpretations, and assessments. The focus of critical thinking is on these thoughtful judgements. The ideal critical thinker is inquisitive by nature, well-informed, trustful of reason, open-minded, flexible, fair-minded in evaluation, honest in the face of “personal biases, prudent in making judgments, willing to reconsider, clear about issues, orderly in complex matters, diligent in seeking relevant information, reasonable in the selection of criteria, focused in inquiry, and persistent in seeking results” that are as precise as the subject and circumstances of inquiry [16, p.3]. As a result, developing effective critical thinkers entails striving towards this goal. It combines the development of critical thinking abilities with the cultivation of attitudes that consistently provide helpful insights and serve as the foundation of a rational and democratic society.

### Critical thinking approach [16]

Facione’s [16] approach proposes six skills, each of which is subdivided into subskills. It involves interpretation, analysis, evaluation, and inference, explanation, and self-regulation [16, p.12]. Interpretation is “to comprehend and express the meaning or significance of a wide variety of experiences, situations, data, events, judgments, conventions, beliefs, rules, procedures or criteria.” [16, p. 13]. Analysis refers to “to identify the intended and actual inferential relationships among statements, questions, concepts, descriptions or other forms of representation intended to express beliefs, judgments, experiences, reasons, information, or opinions.” [16, p. 14]. Evaluation means assessing “the credibility of statements or other representations which are accounts or descriptions of a person’s perception, experience, situation, judgment, belief, or opinion; and assessing the logical strength of the actual or intend inferential relationships among statements, descriptions, questions or other forms of representation.” ([16, p. 15]. Inference is “to identify and secure elements needed to draw reasonable conclusions; to form conjectures and hypotheses; to consider relevant information and to educe the consequences flowing from data, statements, principles, evidence, judgments, beliefs, opinions, concepts, descriptions, questions, or other forms of representation.” [16, p. 16]. Explanation refers to “state the results of one’s reasoning; to justify that reasoning in terms of the evidential, conceptual, methodological, criteriological and contextual considerations upon which one’s results were based; and to present one’s reasoning in the form of cogent arguments.” [16, p. 18]. Self-regulation is “Self-consciously to monitor one’s cognitive activities, the elements used in those activities, and the results educed, particularly by applying skills in analysis and evaluation to one’s own inferential judgments with a view toward questioning, confirming, validating, or correcting either one’s reasoning or one’s results.” [16, p. 19].

The current study relied upon the APA Delphi Consensus Definition of Critical Thinking to specify the areas of critical thinking skills: analysis, interpretation, evaluation, induction, and deduction. Analytical abilities allow people to recognize assumptions, reasons, and assertions, as well as investigate how they interact in the creation of arguments. Analysis is used to extract information from charts, graphs, diagrams, spoken language, and texts. People with high analytical abilities pay attention to patterns and details. They recognize the components of a situation and how those components interact. Strong interpretation abilities can help to assist high-quality analysis by elucidating the meaning of what someone is saying or what something implies. Inference skills show the essential or extremely likely outcomes of a given set of facts and situations. Conclusions, hypotheses, suggestions, or judgments based on flawed analysis, disinformation, poor data, or biased evaluations may turn out to be incorrect, even if they were obtained with strong inference abilities. Evaluative abilities allow us to appraise the reliability of information sources and the assertions they make. These abilities are used to assess the strength or weakness of arguments. We may appraise the quality of analyses, interpretations, explanations, inferences, alternatives, views, beliefs, ideas, proposals, and judgments using evaluation abilities. Strong explanation abilities can help to assist high-quality evaluation by presenting evidence, reasons, techniques, criteria, or assumptions underlying assertions and findings. Inductive reasoning is used to make decisions in ambiguous situations. When we make conclusions about what we think must be true based on analogies, case studies, past experience, statistical analyses, simulations, hypotheticals, and familiar events and patterns of behavior, we employ inductive reasoning abilities. Inductive reasoning exists as long as there is the potential, however distant, that a highly probable conclusion may be incorrect. Inductive reasoning, while not providing certainty, can give a firm foundation for trust in our findings. Deductive reasoning proceeds with exactitude from the presumed reality of a set of beliefs to a conclusion that, if those beliefs are true, cannot be wrong. Deductive validity is strictly logical and straightforward. Unless the meanings of words or the syntax of the language are changed, deductive validity provides no possibility for ambiguity [1].

### Self-study approach to critical thinking

A critical thinking skill, like any other skill, is the ability to do a certain action, process, or operation. In general, having a skill entails doing the correct thing at the appropriate moment. So, being proficient in critical thinking entails understanding, maybe intuitively or without the capacity to describe it, a set of procedures as well as when to employ those methods. Being skillful also entails having some level of expertise in carrying out those processes and being ready to do so when necessary. Reflecting on and developing one’s critical thinking abilities entails determining when one is or is not performing well, or as well as one might, and thinking about strategies to improve one’s performance. Learning critical thinking entails developing the ability to make such self-reflective decisions [16]. Critical thinking abilities may be taught in a variety of methods, including making processes explicit, detailing how they should be applied and implemented, explaining and modeling their right usage, and justifying their use. Teaching critical thinking skills also entails introducing learners to scenarios when the desired processes are justified, rating their performance, and offering constructive feedback on both their competency and strategies to improve it. Instruction may begin with intentionally simple scenarios but should end with ones that are truly complicated. Learners must devote a significant amount of own work, attention, practice, desire, and, when they learn how, self-monitoring, particularly in the case of critical thinking. Teaching skills include encouraging students to reach increasing degrees of competency and, especially in the case of critical thinking, independence. It also entails advising students on how to reach their objectives [16].

Self-study, according to [17], is an approach focusing on teaching and learning experiences that inspire students to see their learning and practice in new ways. Scholars have recognized five features for self-study: the work is self-initiated and targeted; it is oriented at improvement; it is interactive; it employs numerous, mostly qualitative methodologies; and its validity is based on trustworthiness [18]. In self-study, learners can construct new knowledge, find new solutions to problems, recognize new challenges to address, and respond to various contexts [19]. According to [20], self-study learning enables students to develop a foundation for continued education, allowing them to constantly enhance their abilities and be flexible socially and professionally given the need to identify and justify the practical use of techniques for critical thinking which contributes to the development of independent work of students. In this regard, Morozova et al. [20] showed evidence of the connection between critical thinking ability and independent learning; students who learned to think critically boosted their independent learning. According to [21], interactive e-books in scientific education are interactive and may be utilized for self-study to improve students’ critical thinking abilities.

### Teaching critical thinking utilizing technology

Incorporating technology into critical thinking skills training has seen extremely timid attempts. According to [22], the literature revealed that studies rarely attempted to directly explore the effect of new technology, such as websites about teaching and promoting students’ critical thinking, and there is a need for additional research on technology and its impact on fostering critical thinking skills. Past research examined the use of some technologies as mediating tools for teaching critical thinking skills directly and indirectly, such as simulation-based learning [23, 24], Blackboard [25], online discussion [26, 27], WebQuest [28], social networking [29], online social interaction [30], short film clips [31]. The results of those studies indicated the positive impacts of utilizing technologies that boost learners’ critical thinking skills. In the current study, Telegram, a smartphone application, was used to mediate students’ self-study of learning materials in form of videos, discussions, and exercises on cultivating critical thinking skills.

### Critical thinking and learning a foreign language

The talk on integrating critical thinking in foreign language learning started in the 1990s [7]. There were reasons for the need for TESOL educators to implement critical thinking in language education, as argued by Atkinson [6], these reasons indicate critical thinking is a social practice that everybody can learn and practice in any environment unconsciously. Also, it is exclusive and reductive; it reduces all beneficial academic thinking skills. The same notion is supported by [32], who argues that second-language learners require exposure to critical thinking precisely because it is foreign to their cultural patterns of reasoning. Afshar and Movassagh [7] recommended that English teachers deliberately educate EFL learners’ critical thinking abilities, such as reasoning, questioning, deduction, and induction so that the students do not take things for granted and respect for intelligent criticism and direct statement. As a result, second-language instructors have more reasons than first-language teachers to design courses that introduce and expose students to diverse facets of critical thinking. A plenty of studies cited a bilateral connection between critical thinking and foreign language learning. Citing some studies, In Iranian undergraduates’ EFL writing, Nejad et al. [13] revealed a strong association between learners’ critical thinking skills and language learning techniques. Fahim et al. [33] discovered a favorable relationship between students’ critical thinking abilities and their performance on the Test of English as a Foreign Language (TOEFL) reading part. Critical thinking was also linked to ESL writing [34] and total language competency [35]. Khamkhong [11] found that the reading literacy assessment framework increased Thai EFL students’ English critical reading competency and may be utilized as a teaching model for developing the EFL learners’ critical reading and thinking abilities. The students were also pleased with the subject’s new instruction. Chason et al. [9] revealed that critical thinking instruction positively affects L2 learners’ writing quality. According to [8], raising awareness of explicit critical thinking improved Iranian postgraduate TEFL students’ reading comprehension and the correct creation of argumentative essays. According to [15], the infusion strategy increased students’ critical thinking and L2 writing scores significantly, and there was a strong positive association between students’ critical thinking and L2 writing scores. El Soufi and See [10] evaluated existing evidence on the influence of critical thinking teaching on English language learners’ critical thinking skills in higher education. The best proof of effectiveness was found to be explicit education in general critical thinking abilities. According to [12], collaborative writing encouraged Thai EFL students to employ critical thinking abilities while writing. Starichkova et al. [14] revealed that students’ growth of English as a second language can be utilized to successfully create students’ critical thinking abilities. However, there has been research to have cited no correlation between critical thinking and second language learning. For example, Floyd [36] investigated the impact of second-language thinking on critical thinking proficiency. According to the findings, critical thinking achievement is more challenging in English as a second language. According to [37], reading and writing techniques increase student argumentation capacity; nonetheless, they lack critical thinking abilities.

Education, in general, necessities that learners are critical thinkers who do not take every issue for granted; rather, they question and inquire about everything to make decisions and solve problems based on their analysis. Therefore, imparting critical thinking ability in education has received good attention from researchers. Penkauskienė et al. [38] examined critical thinking reflection in Lithuanian higher education programs. The programs were analyzed against nine critical thinking skills. The results indicated that the most embedded critical skills were analysis, evaluation, and decision-making in the course goals and learning outcomes. Explanation, interpretation, and inference skills were not loud enough. Indrašienė et al. [39] sought to reveal the attitude toward the importance of critical thinking in the modern labor market and toward the responsibility for developing it from the perspective of different stakeholder groups in Lithuania. It was found that in both higher education and the labor market, critical thinking is treated as a developed and dynamic competence to encompass both cognitive skills and dispositions. All stakeholder groups consider inference and argumentation the most important critical thinking skills in the modern labor market. Critical thinking dispositions, such as self-confidence and fairness are the most valued. Afshar and Movassagh [7] investigated the relationship between critical thinking, strategy use, and university achievement. The results indicated that both critical thinking and strategy use had significant positive correlations with university achievement. Also, critical thinking was an asset to the high-achieving group. Lailiyah and Wediyantoro [40] described students’ critical thinking attitudes and beliefs in asynchronous learning contexts among English as a Foreign Language (EFL) students at one of Malang’s institutions. The findings revealed that, while the questionnaire analysis revealed students’ good views toward critical thinking skills, the interview revealed students’ lack of confidence. In addition, there existed a multidimensional conception of critical thinking. Van Der Zanden et al. [41] explored how secondary school instructors consider and nurture critical thinking abilities to prepare their pupils for university. It was demonstrated that instructors have a clear vision of critical thinking abilities and that their growth in pupils is entirely dependent on the teacher. According to [42], the case study technique can improve students’ critical thinking skills and assist them in examining and solving problems during discussions in groups among business management undergraduates. Tiruneh et al. [43] investigated the impact of incorporating critical thinking skills education in physics on critical thinking skill development and course performance in studying physics. Participants scored higher on critical thinking skills and course success, according to the findings. Alharbi et al. [44] investigated the influence of an E-collaborative learning environment on critical thinking skill development. The results demonstrated that E-collaborative learning via Blackboard had a substantial and favorable influence on the development of kindergarten-major students’ critical thinking. Hsu [45] showed that collaborative learning promoted learners’ critical thinking development in Engineering Ethics to work harder and think deeper.

After securitizing previous studies, it is evident that cultivating critical thinking among learners is mediated by various approaches, such as collaboration (online/offline), critical thinking instruction, and case study techniques to improve learners’ attainment of critical thinking skills. Also, it was shown that critical thinking ability is a high-demanded skill valued by stakeholders, including educators and employers. In addition, students’ acquisition of critical thinking skills depends considerably on their teachers’ readiness and preparedness. Furthermore, students have a good attitude toward critical thinking skills but lack the confidence to employ them. However, research on cultivating critical thinking skills in Saudi higher education institutions, in general, and the EFL context, in particular, seems very limited. Therefore, the current study attempts to fill this gap by researching the potential of strengthening EFL students’ critical thinking skills through a video-mediated self-study program through Telegram in light of Saudi Vision 2030 and labor market needs. Therefore, this research attempts to investigate learners’ attainment level of critical thinking skills pre-and post-utilization of a video-mediated self-study program through Telegram to enrich EFL learners’ critical thinking skills to answer the following research questions:

1. What is the impact of a video-mediated self-study program through Telegram on EFL students’ critical thinking skills?
2. What are students’ learning experience of critical thinking skills using a video-mediated self-study program through Telegram?

### Conceptual framework

The conceptual framework of the present study is informed by Critical thinking approach by Facione [16], self-study approach, and video-mediation technology. The critical thinking approach proposes six skills, each of which is subdivided into subskills. It involves interpretation, analysis, evaluation, and inference, explanation, and self-regulation. Facione’s [16] critical thinking approach was taught using self-study approach, focusing on enabling students to develop a foundation for continued education, allowing them to constantly enhance their abilities and be flexible socially and professionally given the need to identify and justify the practical use of techniques for critical thinking which contributes to the development of independent work of students [17]. In addition, Telegram was used to mediate students’ self-study of critical thinking learning materials in form of videos, discussions, and exercises on cultivating critical thinking skills. Moreover, EFL students’ attainment of critical thinking skills as specified by Insight Assessment [1] was measured post the instructional program.

## Methodology

### Research design

The study employed the quasi-experimental approach by the time series design with a pre-and post-treatment group. The quasi-experimental design with only one treatment group has various names, such as time-series design and pre-post treatment, and pre-post study design. This design involves repeated observations (both pre-test and post-test) over a set period. Before the treatment, a set of observations is made to establish a baseline. Following the treatment, further observations are made to ascertain the effects of the treatment [46]. The current study employed a pre-and post-test and a semi-structured interview to collect the data from undergraduates at the College of Languages and Translation at Najran University in the Kingdom of the Saudi Arabia in the third semester of the academic year 2023.

### Population and sample of the study

Undergraduates majoring in the English language at the College of Languages and Translation at Najran University are the intended participants of the study. One section totaling (no = 30) students was chosen in the treatment group who learned critical thinking skills using a video-mediated self-study program through Telegram. The intended students are in the ninth, tenth, and twelfth levels. They self-studied the learning material in form of videos sent via Telegram. Both male and female students who participated in the study are in the 22–23 age group. Over eleven years, they have been exposed to English studies, including school and university. Therefore, their English level should be considered upper-intermediate. This level suggests that they initiate conversations, make discussions, raise inquiries, and express their opinions about what they read and watch the instructional material. An approval letter for conducting the research from the Ethical Approval Committee at the Deanship of Scientific Research, Najran University was obtained with the code [009773-021280-DS] on 29/03/2023. Besides, all the necessary approval letters were obtained from the ethical committee at Najran University, the context of the study. Also, the participants’ written consent forms were collected after being signed by the students who agreed to participate in the study voluntarily. The recruitment period for the study started on April 1, 2023 and ended on June 13, 2023.

### Study instruments

The researchers used a critical thinking test (the California Critical Thinking Skills Test (CCTST) and a semi-structured interview to collect data from the participants’ learning of critical thinking skills using a video-mediated self-study program via Telegram before and after the experiment. While the test was used to measure the participants’ level of improvement pre- and after post-the experiment acquiring critical thinking skills, the semi-structured interview will collect data on their learning experience and attitude toward critical thinking skills using a video-mediated self-study program via Telegram.

After reviewing the literature review, the researchers decided to implement a modified version of the California Critical Thinking Skills Test (CCTST) (2000) because of its suitability for university students. CCTST is an educational assessment that measures all the core reasoning skills needed for reflective decision-making. It provides valid and reliable data on individuals’ and groups’ critical thinking skills. It is designed for use with undergraduate and graduate students. It is available in many languages, and its overall skills score can be benchmarked using one of many percentile comparisons. It is most commonly used for admissions, advising and retention, studies of curriculum effectiveness, accreditation, and the documentation of student learning outcomes. The test had some modifications to fit the Saudi Arabian context of the current study and the importance of these skills by university graduates and their alignment with the Saudi labor market proposed in the Saudi Vision 2030. The test measures the critical thinking scores of the study sample. The test items consist of (34) items divided into five sub-skills of critical thinking skills. The analysis skill refers to accurately identifying the problem and decision-critical elements and has six items. The evaluation skill assesses the credibility of claims and the strength of arguments and includes six items. The inferencing skill draws warranted and logical conclusions from reasons and evidence and contains four items. The deduction skill is a reasoned judgment in precisely defined, logically rigorous contexts and includes 12 items. The induction skill refers to reasoned judgments in ambiguous, risky, and uncertain contexts and has six items [1]. Concerning the CCTST time, the average period for completing the test is 45 minutes. The test-takers who complete the test in less than 15 minutes should be discarded due to the unrepresentativeness of the actual performance of the test skills. Table 1 shows an adapted scoring version from CCTST Test Manual to be used in the current study [1].

**Table 1:** Recommended performance assessments for the CCTST score.

Recommended performance assessments overall scores	CCTST overall score–recommended performance assessment				
	Not manifested	Weak	Moderate	Strong	Superior
**CCTST overall score 34-point form 2000 versions**	0–7	8–12	13–18	19–23	24–34
**Recommended performance assessments (skill-wise)**	CCTST skill-wise score–recommended performance assessment				
	Not manifested	Moderate		Strong	
**Analysis skill**	0–2	3–4		5–6	
**Inference skill**	0–1	2–3		4	
**Evaluation skill**	0–2	3–4		5–6	
**Induction skill**	0–2	3–4		5–6	
**Deduction skill**	0–5	6–11		12	

### Validity and reliability

The CCTST test is a worldwide standardized test. It is an educational assessment that measures all the core reasoning skills needed for reflective decision-making. It provides valid and reliable data on the critical thinking skills of individuals and groups. The instrument development team includes experts in critical thinking, assessment, psychometrics and measurement, statistics, and decision science. Continuing research on the CCTST focuses on the valid and reliable measurement of testing critical thinking skills at all levels of educational and occupational expertise. Evidence for the construct validity of the CCTST is provided by the demonstration of improvement in students’ CCTST test scores after they have taken a course in critical thinking or an educational program training on the critical thinking portion of clinical reasoning. Past studies cited from many countries all over the world that used the CCTST documented gains in critical thinking skills. The data from ongoing validation studies produce internal consistency estimates (Kuder Richardson-20) ranging from .68-.80. The Kuder-Richardson-20 is used for dichotomously scored instruments and scales for instruments with multidimensional scales. Seventy percent indicates a high level of internal consistency [1]. In the current study, the researchers applied the test to an exploratory sample of (30) students from the College of Languages and Translation in the English Language and Translation Departments. Couder-Richardson reliability coefficient values-20 were calculated on the skills and the total score of the test. The analysis results showed that the coefficient of reliability according to Codder-Richardson-20 was (.88). This value is considered high and indicates the test reliability.

### Instructional program

Instructional design is the methodological process of transforming learning and instruction specifications into strategies for instructional resources, behaviors, information materials, and assessment. It is a procedure that produces, develops, and disseminates learning materials and experiences. These resources include but are not limited to online courses, instructional materials, videos, and other learning simulations. This study intends to adopt a video-mediated self-study program via Telegram where the students watch videos and interact with their peers and experienced teachers on learning materials related to boosting critical thinking skills. The learning materials were covered outside the classroom. Telegram is a free, partially open-source, cross-platform instant messaging application focusing on security. Telegram users can exchange messages with high encryption capabilities, including photos, videos, and documents, as all files are supported. It was founded in 2013 by Pavel Valeryevich Durov. The application is available in 58 languages.

The instructional program for learning critical thinking skills is based on the concepts of online learning via Telegram (delivery of learning materials), self-study, and interaction with peers and researchers. Online learning of critical thinking skills was resorted to because these skills are not graded, and online learning of them would encourage students to learn them as it saves time, effort, and cost, and access to the learning materials at any place and time. Students used the self-study method to learn critical thinking skills through watching interactive videos on critical thinking. The videos included all the materials students needed to learn how to think critically. Finally, interactive learning was included in the student’s interaction with their peers and teachers via the Telegram group and the videos which included some interactive learning materials that gauge students’ thinking. The instructional program included three main phases: pre, during, and post. Before starting the program, the intended participants were briefed about the study objectives, data collection tools, and their roles in the study, and collected their signed consent forms. Also, all inquiries coming from the participants were answered by the researchers. In the same session, the researchers administered the pre-test of the CCTST on campus. During the treatment, the researchers held an online orientation session via Blackboard, in which the participants were briefed about the definition, skills, importance, and qualities of critical thinking. Also, they were oriented on the steps of implementing the program via Telegram, the self-study learning materials, and the teacher’s role in students’ engagement. After that, the researchers created a group on Telegram and added all the participants. In that group, they received videos on critical thinking from various sources on the Internet, such as YouTube and teachers’ self-development. They had to watch the videos and inquire about points that need clarification in the Telegram group. The students and researchers answer their inquiries. The videos were classified according to the five critical thinking skills: analysis, evaluation, inference, induction, and deduction. An average of one to two videos were on each skill. Students watched the videos and interacted with their peers and researchers. Post watching and interacting with each video, the participants practiced exercises related to each critical thinking skill. The treatment lasted for five weeks: Every week, the participants learned and practiced one critical thinking skill. Post the training program, the participants sat for the post-test of the CCTST followed by a semi-structured interview with those who agreed to the interview on campus.

### Data analysis

To answer research question 1, the means of the pre-and post-test of the CCTST were calculated to compare the impact of the training program of critical thinking skills on students’ level of attainment of these skills. Also, the paired-sample t-test was used to compare the pre-and post-test results for the same group (treatment group). Couder-Richardson equation 20 was applied to detect the test reliability. The t-test for paired samples was computed for any statistically significant differences before and after the treatment. Effect size using Cohen’s [47] equation was used to calculate the effectiveness of the video-mediated self-study program via Telegram on EFL students’ development of Critical thinking skills: (effect size = (t)/square root of the sample). A semi-structured interview, using protocols developed by the researchers, was employed to answer research question 2, about the participants’ learning experience of critical thinking skills using the video-mediated self-study program via Telegram. In answering the second research question, the participants were required to express whether they liked videos and Telegram as communication tools and make suggestions concerning how their critical thinking skills could be improved in terms of learning materials, teaching methods, video content, communication tools, and activities. The qualitative data from the semi-structured interview were content-analyzed based on repeated occurrences. They were grouped by the researchers under main themes.

## Study results

### The impact of a video-mediated self-study program on EFL students’ critical thinking skills

Table 2 shows the results of the t-test for paired samples on the skills and total score of the critical thinking skills test before and after the intervention. There were significant differences at (0.05) in the participants’ scores in the pre-and post-test of critical thinking skills. The significance came in favor of the participants’ post-performance (t(29) = -2.605-, p>.05). This result means that the intervention was slightly effective (effect size = 0.47). That is to say, the students improved their development of critical thinking skills due to the training program compared to their performance before the treatment at a low level. Concerning critical thinking skills, the results showed significant differences in the participant’s scores in the skills of analysis, inferencing, and deduction; they medially or slightly developed these skills compared to their performance before the intervention. However, they failed to improve their scores in the post-test in the skills of induction (t(29) = -1.439-, p < .05) and evaluation (t(29) = .701, p < .05).

**Table 2:** Paired samples t-test results (critical thinking skills pre- and post-test).

Vocabulary	Test	Mean	Std. Deviation	t	df	Sig. (2-tailed)	Effect size	Level—Effect size
**Analysis**	Pre-test	2.60	.621	-3.084-	29	.004	0.56	Medium
	Post-test	3.17	.592					
**Induction**	Pre-test	2.97	.669	-1.439-	29	.161	-	-
	Post-test	3.17	.648					
**Inferencing**	Pre-test	2.40	.563	-2.163-	29	.039	0.39	Low
	Post-test	2.73	.583					
**Deduction**	Pre-test	2.60	.563	-3.003-	29	.005	0.54	Medium
	Post-test	2.97	.490					
**Evaluation**	Pre-test	2.63	.556	.701	29	.489	-	-
	Post-test	2.57	.504					
**Total score of the test**	Pre-test	13.37	1.351	-2.605-	29	.014	0.47	Low

### Students’ learning experience of critical thinking skills using a video-mediated self-study program through telegram

The interviewees’ answers to the questions in the semi-structured interview were recorded and analyzed. The analysis results revealed a number of themes that the participants’ answers revolved around. The themes are the way of thinking, feelings, benefits, motivation, challenges and problems, and suggestions. Concerning the participants’ way of thinking, all the interviewees noted that their way of thinking has changed after the intervention in terms of looking at things from different perspectives, patience in making decisions, judging things, understanding things, and using given information to reason well as shown in the following excerpts:

S1: “Yes, my mind has another horizon”
S2: “Kind of, by analyzing and not rushing and deliberating in answering”
S4: “Yes, I can now differentiate between the things that support my thinking”
S6: “Yes, a little, in understanding the question and the way to think about the answer”
S9: “My thinking changed in terms of checking and deducing the answer with all logical inferences”
S19: “Yes, it differed, but not much. The difference was in the method of analysis, thinking and issuance of judgments, but the difference was partial and not entirely”

As for the participants’ feelings about learning critical thinking skills using Telegram-mediated learning materials, the absolute majority of the participants remarked that they felt positive because of the easy access to information, interaction, the importance of the topic, time and effort, learning new things, and change in the thinking ways. The following support these feelings:

S2: “Emotions are very positive and are evident in the interaction with critical thinking tests. Negative ones are not in my dictionary”
S5: “It was short and saved effort, time, and money”
S9: “Positive in terms of learning a new skill and negative in terms of intellectual effort”
S18: “Positive feelings in trying to think of more than one solution and choosing the most comprehensive answer”
S19: “The positive feelings were in acquiring new things and knowing some things, and the negative ones were my ignorance about some things that I did not know”
S21: “I had negative feelings coz I did not know how critical thinking is, and after I learned critical thinking, I became positive even in my thinking”

Upon asking the interviewees about the benefits they gained from the critical thinking program mediated by Telegram, they confirmed that their thinking improved in terms of thinking differently, learning to assess well before making the final decision, acquiring highly-demanded skills for the labor market, reaching conclusions, using mind instead of heart when thinking, and thinking deeply. The following are some examples of the participants’ answers:

S2: “Analysis, thinking, searching for the answer, and choosing the answer after analyzing all the answers, specifically which ones are closest to the truth”
S4: “Refine your thinking and change your perception of information that was wrong”
S9: “I benefited from not rushing to answer, I also benefited from thinking out of the ordinary, and I benefited from innovation in information”
S19: “I benefited from how to analyze and think, and in the end how to deduce the outputs”
S21: “It improves your ability to use your mind instead of your emotions, and you can identify your feelings and link them logically with your thoughts, and this helps you develop better levels of thinking”
S24: “Deep thinking behind the questions”

Based on the interview, the participant explained their motivation for learning critical thinking skills. Almost all of them mentioned that the program motivated them to learn critical thinking due to easy access to learning materials, widening horizons in thinking, learning to solving problems in everyday life, being wise to make the right decision, and simple thinking and understanding things. Following are some excerpts from the participants’ answers:

S1: “Yes, because it is easy and I can learn it at any time”
S2: “Yes, as seeing the clips via telegram and benefiting from them was a great motivation and a catalyst for expanding perceptions towards criticism and thinking seriously”
S5: “Yes, it was motivating and helping to behave properly in dangerous situations and you should think carefully”
S6: “Yes, it helped me in the way of understanding and simplification”
S9: “Yes, it was stimulating and of high quality in terms of interest in my answer”

Regarding challenges and problems of learning critical thinking skills, the participants referred to the shortage of time, the difficulty of the topic, understanding critical thinking, and changing one’s thinking habits. The following are some excerpts from the interviewees’ answers:

S2: “The lack of time for me due to my late arrival, but there is difficulty, as the questions did not cancel the pleasure despite the difficulty”
S10: “Changing ways of critical thinking and clarifying it”
S17: “Challenge of intelligence and relying on intuition”
S19: “I faced some problems, including how to count the data and extract the result from it, but I feel that I have improved a little”
S21: “Stereotypical or emotional thinking that favors the familiar over the unfamiliar, the familiar over the strange and the known over the unknown. This may put the individual in a circle of misperceptions that negatively affect his personal and professional life.”

Finally, the participants proposed some solutions for overcoming the challenges and problems they face in learning critical thinking skills, such as intensifying the program, using more interactive videos, increasing the learning materials on critical thinking, repeating the experiment, and using group-based games in improving critical thinking. The following are some excerpts from the interviewees’ answers:

S2: “Intensifying such experiments and assessing the level of students”
S3: “Asking clips containing interactive questions”
S5: “Continue in this way and I think it will contribute to the development.”
S19: “I suggest increasing in educational topics regarding critical thinking”
S21: “There are ways to develop critical thinking skills and have easy and simple activities, such as group games”.

## Discussion

The results of the quantitative data showed that the participants improved their learning of critical thinking skills based on the intervention; however, the effect size was low. That is to say, the students improved their critical thinking skills due to the training program compared to their performance before the treatment at a low level. The participants self-studied the learning materials sent to them via Telegram to improve their critical thinking skills. Therefore, the video-mediated self-study strategy was found beneficial in assisting students’ enhancement of learning critical thinking skills. Students self-studied critical thinking learning materials in form of videos, discussion, exercises, and feedback. This result indicates that learning critical thinking must be developed, practiced, and continuously integrated into the curriculum to engage students in active learning. Video is a rich and powerful media used in learning critical thinking skills. It can present information in an interesting way. Interactive video improves learning interactivity, thus potentially improving students’ effectiveness and student motivation consistently. Also, video allows students to learn by themselves by evaluating and reflecting on their critical thinking learning and motivates them to learn better by providing direct feedback and reinforcement in a fun and exciting atmosphere [48, 49]. In this regard, Ambarwati and Suyatna [21] assert that the use of videos in education has a positive effect to increase students ’attention and curiosity and help provide in conceptual learning, so as to enhance students’ critical thinking skills. Also, Ambarwati and Suyatna [21] opined that interactive e-books in scientific education can be utilized for self-study to improve students’ critical thinking abilities.

The weak result of learning critical thinking skills from the quantitative data in the current research can be attributed to the difficulty of critical thinking skills as they require more time, effort, and practice from learners. Also, maybe the program implemented in the current study was not efficient since it lasted only for ten weeks. Likewise, the qualitative data showed that the participants referred to the shortage of time, the difficulty of the topic, the understanding of critical thinking, and changing one’s thinking habits. In addition, the program was delivered remotely via Telegram as a result the participants might not have taken it seriously and thus had not self-studied the learning materials thoroughly. Moreover, learning these skills was not part of any summative assessment; and was not graded, so the participants did not pay much attention to them; nevertheless, they expressed their motivation and enthusiasm in taking this experiment learning critical thinking as expressed in the interview. Furthermore, university curricula and tests are not designed in a way that encourages critical thinking; students did not have any background about the topic before as teachers rarely use critical thinking in teaching English subjects. Smith and Szymanski [50] argue standardized testing methods have made students to rely on rote learning and recall to improve test scores, which leaves little time to focus on teaching higher-level thinking skills. Finally, the participants learned critical thinking skills in English, and this added more burden on their thinking process. Floyd [36] claims that critical thinking achievement is more challenging in English as a second language. He argues students’ critical thinking had better scores when taking the critical thinking test in their mother tongue (Chinse) than English.

In addition, the result supports that of Morozova et al.’s [20] study, which showed evidence of the connection between critical thinking ability and independent learning; students who learned to think critically boosted their independent learning. Besides, the current result echoes that of Zuluaga et al. [37], who showed that reading and writing techniques increase students’ argumentation capacity; nonetheless, they lack critical thinking abilities. Moreover, Al-Fneikh [51] unveiled that the critical thinking level is low among Qassim University students. Similarly, Bataineh and Zghoul [52] identified that TEFL graduates performed quite poorly on the critical thinking skills test. However, the result is somewhat inconsistent with that of Alharbi et al. [44], who demonstrated that E-collaborative learning via Blackboard had a substantial and favorable influence on developing kindergarten-major students’ critical thinking. The difference in the results can be attributed to the nature of the experiments and students’ majors.

Concerning critical thinking skills, the results showed significant differences in the participant’s scores in the skills of analysis, inferencing, and deduction; they medially or slightly developed these skills compared to their performance before the intervention. These results can be attributed to the nature of the three skills; the participants, maybe, did better in these skills as they are relatively easier than the induction and evaluation skills. Also, these skills are addressed in the daily life of individuals and the curricula in schools and universities indirectly to some extent. They need them to know causes and effects by generating arguments and assumptions and looking for evidence. However, they failed to improve their scores in the post-test in the skills of induction and evaluation. The result is due to the fact that these two skills are not easy to comprehend and need more and more learning and practice. Especially, the evaluation skill comes as the last learning skill and thus requires a lot of thinking and information so that students can justly evaluate things. In addition, the induction skill includes the indications and judgments that a person makes after referring to a situation or event. Also, the skill of evaluation involves evaluating claims and arguments. In addition, the difficulty comes from the weakness of the individual’s practice of these operations in life and the weakness of teachers’ possession of these processes and their use in classroom situations.

The findings of the qualitative data showed that all the interviewees noted that their way of thinking has changed after the intervention in terms of looking at things from different perspectives, patience in making decisions, judging and understanding things, and using information to reason well. In this regard, Dwyer et al. [53] argues that instruction in critical thinking is prior to learning as it can improve students’ understanding of information and cultivate better judgment in their everyday life. Also, the participants felt positive because of the easy access to information, interaction, the importance of the topic, time and effort, learning new things, and change in their thinking ways. In line with this finding, Lailiyah and Wediyantoro [40] showed that Indonesian EFL students have positive attitudes toward confidence in their critical thinking. In addition, the interviewees admitted that the program motivated them to learn critical thinking due to easy access to learning materials, widening horizons in thinking, learning to solving problems in everyday life, being wise to make the right decision, and simple thinking and understanding things. Finally, the participants proposed some solutions for overcoming the challenges and problems they face in learning critical thinking skills, such as intensifying the program, using more interactive videos, increasing the learning materials on critical thinking, repeating the experiment, and using group-based games to improve critical thinking.

Based on the findings of the current study, it is suggested that the video-mediated self-study strategy assists in boosting EFL students’ critical thinking skills. Their thinking improved in terms of thinking differently, learning to assess well before making the final decision, acquiring highly-demanded skills for the labor market, reaching conclusions, using the mind instead of heart when thinking, and thinking deeply. Accordingly, the video-mediated self-study strategy can help students learn critical thinking due to easy access to learning materials, widening horizons in thinking, learning to solving problems in everyday life, being wise to make the right decision, and simple thinking and understanding things.

## Conclusion

This research experimented with a video-mediated self-study program through Telegram on learning critical thinking skills for EFL students majoring in English or Translation. The findings showed that students slightly improved their critical thinking skills in analysis, inferencing, and deduction; however, they failed to improve their induction and evaluation skills based on the quantitative data. Also, the qualitative data revealed that the participants had positive attitudes toward learning critical thinking skills, and the implemented program motivated them to think critically via a video-mediated self-study program. The study implicates that university students can make of self-studied learning materials and collaborative learning methods mediated by technology to think critically with inventive thinking since critical thinking, self-study, and learner autonomy are of high priority in today’s education and the labor market needs. The study was limited to EFL graduates at a Saudi university; therefore, the results may not be generalizable to other contexts. Also, the program lasted for ten weeks which may not be enough to promote critical thinking skills at the required level. The interventional program was delivered online through Telegram, so it was difficult to trace students’ learning of critical thinking materials. In light of the current findings, teachers should foster critical thinking in their EFL courses by incorporating chances for their students to read, write, and discuss. Also, the Ministry of Education should conduct critical thinking workshops for students and instructors, which will benefit students, especially if the workshops are designed for the learning context and students’ needs and learning styles. In addition, universities should impose a compulsory subject on critical thinking for all universities since critical thinking is a highly-demanded skill in the labor market. Furthermore, study plans should be revised concerning the inclusion of critical thinking skills in their subjects as well as teaching methods. Future studies may focus on utilizing games that motivate students to think creatively in improving learners’ critical thinking skills among EFL learners. Also, interactive learning materials that gauge EFL students’ thinking can be examined.

## Supporting information

S1 Dataset (SAV)
